# Cultivation of the Dematiaceous Fungus *Cladosporium sphaerospermum* Aboard the International Space Station and Effects of Ionizing Radiation

**DOI:** 10.3389/fmicb.2022.877625

**Published:** 2022-07-05

**Authors:** Nils J. H. Averesch, Graham K. Shunk, Christoph Kern

**Affiliations:** ^1^Department of Civil and Environmental Engineering, Stanford University, Stanford, CA, United States; ^2^Center for the Utilization of Biological Engineering in Space, Berkeley, CA, United States; ^3^Physics Department, North Carolina School of Science and Mathematics, Durham, NC, United States; ^4^Higher Orbits “Go for Launch!” Program, Leesburg, VA, United States; ^5^Department of Statistics, Ludwig Maximilian University of Munich, Munich, Germany; ^6^School of Social Sciences, University of Mannheim, Mannheim, Germany

**Keywords:** space radiation, micro-fungi, *Cladosporium sphaerospermum*, radiotrophy, biotechnology, *in situ* resource utilization

## Abstract

In Space, cosmic radiation is a strong, ubiquitous form of energy with constant flux, and the ability to harness it could greatly enhance the energy-autonomy of expeditions across the solar system. At the same time, radiation is the greatest permanent health risk for humans venturing into deep space. To protect astronauts beyond Earth's magnetosphere, advanced shielding against ionizing as well as non-ionizing radiation is highly sought after. In search of innovative solutions to these challenges, biotechnology appeals with suitability for *in situ* resource utilization (ISRU), self-regeneration, and adaptability. Where other organisms fail, certain microscopic fungi thrive in high-radiation environments on Earth, showing high radioresistance. The adaptation of some of these molds to areas, such as the Chernobyl Exclusion Zone has coined the terms positive “radiotropism” and “radiotrophy”, reflecting the affinity to and stimulation by radiation, and sometimes even enhanced growth under ionizing conditions. These abilities may be mediated by the pigment melanin, many forms of which also have radioprotective properties. The expectation is that these capabilities are extendable to radiation in space. To study its growth in space, an experiment cultivating *Cladosporium sphaerospermum* Penzig ATCC® 11289™ aboard the International Space Station (ISS) was conducted while monitoring radiation beneath the formed biomass in comparison to a no-growth negative control. A qualitative growth advantage in space was observable. Quantitatively, a 1.21 ± 0.37-times higher growth rate than in the ground control was determined, which might indicate a radioadaptive response to space radiation. In addition, a reduction in radiation compared to the negative control was discernable, which is potentially attributable to the fungal biomass.

## Introduction

### Background

With concrete efforts to send humans to the Moon by 2024 under the Artemis program and establish a permanent presence on the next rock from Earth by 2028, humankind looks to Mars as the next big leap in space exploration (NASA, 2020). In preparation for prolonged human exploration missions venturing beyond Earth-orbit and deeper into space, the required capabilities increase significantly (Loff, 2018). While advanced transportation solutions led by the public and private sectors alike (e.g., SLS/Orion, Starship, New Glenn) are pivotal and have already reached high technological readiness, resource-management and life-support systems, as well as crew health and performance, are equally essential. Therefore, any mission scenario [cf. “Design Reference Architecture 5.0” (Drake and Watts, 2014) or “Mars Base Camp” Cichan et al., 2017)] must include innovative solutions that can meet the needs and address the hazards of prolonged residence on celestial surfaces. In particular, meeting energy demands is critical to extending the duration of mission scenarios; proposed solutions have included nuclear power or photovoltaics (Abel et al., 2021). However, both are significant with respect to the up-mass requirements of the hardware. The storage of energy in the form of electricity is further limited by the weight of batteries. Therefore, chemical energy carriers that are based on *in situ* resources would be pivotal.

*In situ* resource utilization (ISRU) is being seen as an essential concept to extend capabilities without penalizing or sacrificing redundancy, as well as to break the supply chain from Earth, which is pivotal to sustaining human exploration of deep space (Hall, 2017). For ISRU, biotechnology holds some of the most promising approaches (Menezes et al., 2015; Rothschild, 2016; Verseux et al., 2016; Nangle et al., 2020; Berliner et al., 2021a). Fundamentally, the ability of biology to fix inorganic carbon can generate carriers of energy and substrates for biotechnology in the form of biomass, which can be converted into more reduced carbon feedstocks, e.g., methane (Averesch, 2021).

Earth's global carbon cycle relies on sunlight—non-ionizing electromagnetic (wave) radiation of the visible spectrum—to fix inorganic carbon. Ionizing radiation, a much more energetic form of radiation, mostly originates from nuclear decay (on Earth's surface and below) and is comparatively rare. In space, ionizing radiation is ubiquitous and consists mostly of energetic particles, two types of which are primarily encountered on celestial bodies like Earth's Moon or Mars: Solar Energetic Particles (SEPs) and Galactic Cosmic Rays (GCRs). These high-energy particles in the range of hundreds of MeV/nuc to GeV/nuc can penetrate deep into the regolith, where they produce secondary particles, including neutrons and γ-rays (Hassler et al., 2014). This radiation environment presents a significant and constant energy flux independent of most environmental factors (unlike sunlight, which can be intermittent due to dust storms on Mars)—being able to harness (parts of) this energy would provide a steady source of energy.

Nevertheless, radiation is also the greatest threat to the short- and long-term health of astronauts on long-duration deep-space missions (Cucinotta et al., 2012; Chancellor et al., 2014; Afshinnekoo et al., 2020). Over the time of one year the average person on Earth is dosed with about 6.2 mSv (ANS, 2020; NRC, 2021), while the average astronaut on the International Space Station (ISS) is exposed to an equivalent of ~144 mSv (Cucinotta et al., 2008); one year into a 3-year mission to Mars, an astronaut would already have accumulated some 400 mSv, primarily from Galactic Cosmic Radiation (GCR) (Letaw et al., 1988). While the particular health effects of radiation exposure on interplanetary travel have not been fully assessed (Chancellor et al., 2018), it is clear that adequate protection against cosmic radiation is crucial for missions beyond Earth orbit; however, solutions are more restricted by up-mass limitations than any other factor of space travel (Ambroglini et al., 2016).

Among all domains of life, there exist extremophiles that live and persist in highly radioactive environments, including bacteria, fungi, and higher organisms, such as insects (Krisko and Radman, 2013; Kalawate and Mehetre, 2015). Further, certain microscopic fungi that populate the Chernobyl Exclusion Zone and Nuclear Power Plant, where radiation levels are 3–5 orders of magnitude above normal background levels, show a phenomenon called “positive radiotropism” as ionizing radiation becomes an orienting factor for these molds (Zhdanova et al., 2004; Karpenko et al., 2006; Belozerskaya et al., 2010). Additionally, some of these dematiaceous fungi have also been found to populate the interiors of spacecraft in low Earth orbit (LEO), where exposure to ionizing radiation is also intensified (Dadachova and Casadevall, 2008). Black molds and their conidia have been found to remain viable even after exposure to an equivalent radiation dose of several months' worth of space radiation (Cortesão et al., 2020); radiation even appears to stimulate their germination (Tugay et al., 2006). How these organisms protect themselves from radiation damage has been the subject of intense study, and specifically, melanin has been explored as a biotechnological means for radioprotection (Pacelli et al., 2017; Malo et al., 2018). As an antioxidant, melanin appears to play a major role in the “radioadaptation” of these molds (Tugay et al., 2011; Eisenman and Casadevall, 2012; Pacelli et al., 2017; Malo et al., 2018). The pigment has also been the subject of investigation as a potential mediator in the transfer of energy from radiation onto metabolism (Dadachova et al., 2007; Dadachova and Casadevall, 2008; Turick et al., 2011). Based on the observed “radiostimulation” of melanogenesis as well as the growth advantage that putatively results from the exposure of these molds to high radiation, the concept of “radiosynthesis” has been developed. If found veritable, melanin could be perceived as loosely analogous to chlorophyll in photosynthesis.

### Concept

Here, the dematiaceous melanotic micro-fungus *Cladosporium sphaerospermum* Penzig ATCC® 11289™ (cf. Supplementary Information 1, Section A) was cultivated in LEO on the ISS. The preference of certain *C. sphaerospermum* isolates for environments with extreme radiation levels on Earth is well documented (Zhdanova et al., 2004; Ledford, 2007; Dadachova and Casadevall, 2008). Consequently, it has been hypothesized that similar proliferation occurs in response to the high radiation environment off-Earth and that such melanized fungi can be utilized for radioprotection in space (Cordero, 2017; Pacelli et al., 2017). The objective of this experiment was to conduct a proof-of-principle study on a single payload, utilizing basic flight hardware for an autonomous experiment in the unique radiation environment of the ISS. This offered the opportunity to test the mold's (growth) response in space while monitoring radiation levels against a no-growth negative control to assess its potential to serve as a bioregenerative means for radioprotection.

## Materials and Methods

### Experimental Setup

Space Tango (Space Tango, Inc., Lexington, KY, US) was contracted for experimental design and construction (terrestrial logistics and on-orbit operations) (CASIS, 2020). The initial concept for the experimental design was adapted by Space Tango for assembly of the flight hardware and implementation aboard the ISS within TangoLab™ facilities. The flight hardware was housed in a 4″ × 4″ × 8″ double unit standard-size CubeLab™ hardware module and consisted of the following main components: two Raspberry Pi 3 Model B+ (Raspberry Pi Foundation, Caldecote, Cambs., UK) single-board computers, EP-0104 DockerPi PowerBoard (Adafruit Industries, New York, NY, US), PocketGeiger Type5 (Radiation Watch, Miyagi, JP) with the PIN photodiode X100-7 SMD (First Sensor AG, Berlin, DE), Raspberry Pi Camera v2 (Raspberry Pi Foundation, Caldecote, Cambridgeshire, UK) light source (0.8 W LED-strip) for imaging, DHT22 integrated environmental sensor suite (Aosong Electronics Co. Ltd, Huangpu District, Guangzhou, CN) for temperature and humidity readings, a real-time WatchDog™ timer (Brentek International Inc., York, PA, US), and D6F-P0010A1 (Omron Electronics LLC, Hoffman Estates, IL, US) electronic flow-measurement system. One Raspberry Pi (“auxiliary-computer”) running Raspbian v10.18 was dedicated to photography, lighting, temperature, humidity, and electronic flow measurement (EFM) readings, while the second Raspberry Pi (“flight-computer”) controlled radiation measurements, stored in a probed Logger Memobox (Fluke Corporation, Everett, WA, US). The assembled flight hardware was calibrated and vetted before flight; in particular, the consistency of the two radiation sensors was confirmed so that no apparent deviation in recorded ionizing events existed between them.

**Figure 1:**
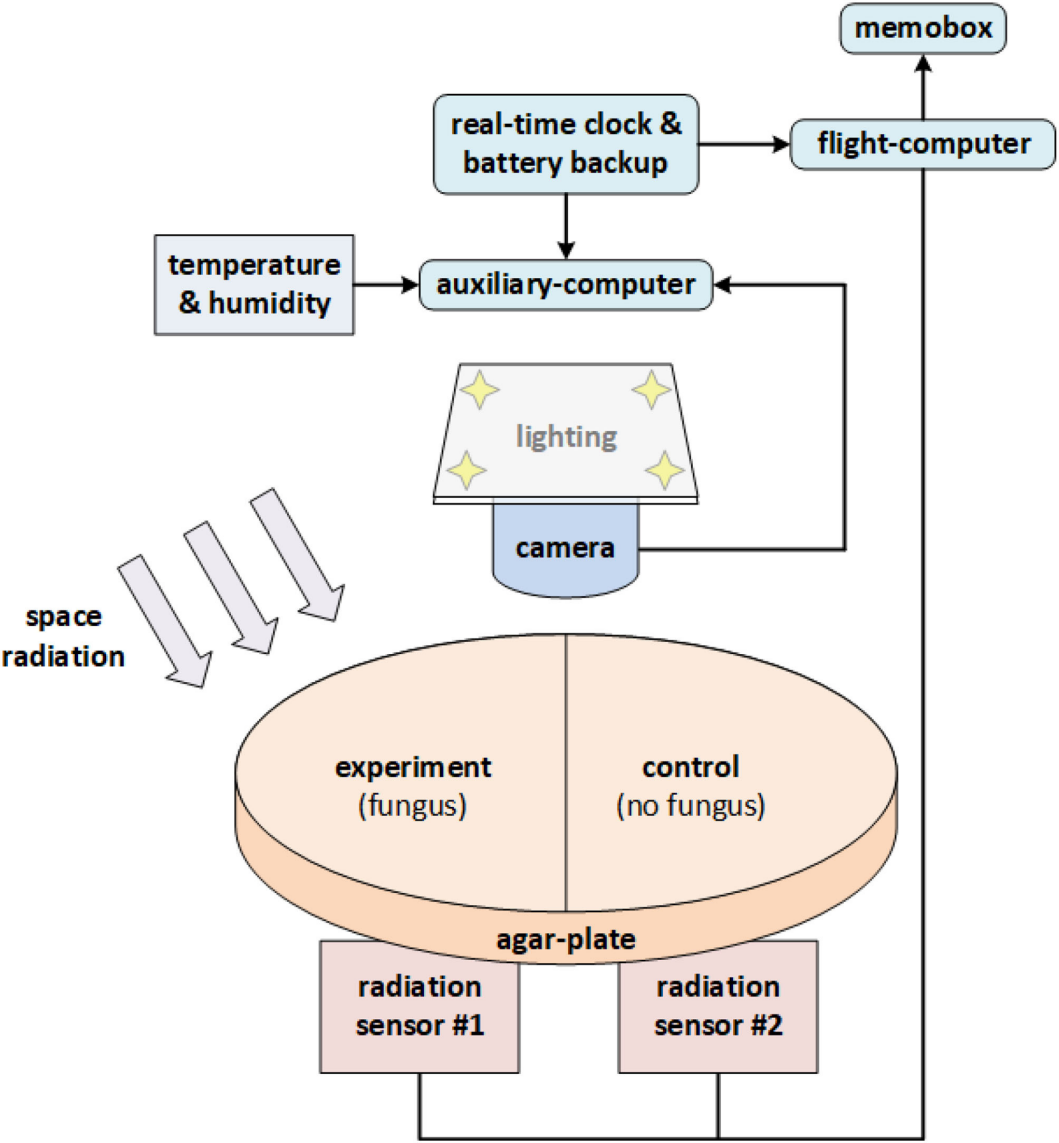
Block chart of the experimental flight-hardware setup. The single (split) Petri dish accommodated both the experiment (agar + fungus), as well as the negative control (agar only). The two (square) radiation sensors were situated in parallel directly beneath the Petri dish (one for each side). Note that “shielding” is one-sided only (for simplicity of experimental setup and to reduce mass).

*Cladosporium sphaerospermum* (ATCC® 11289™, strain designation CBS 2 [CBS 193.54, IMI 49637]) was obtained from Microbiologics® (St. Cloud, Minnesota, US), catalog no. 01254P (supplied as KWIK-STIK™). The growth medium was potato dextrose agar “PDA” (Carolina Biological, Burlington, NC, US) obtained as a “Prepared Media Bottle” (approx. composed of 15 g/L agar, 20 g/L glucose, and 4 g/L starch). A total of 20 ml of PDA (dyed with orange 1) was used to fill the two compartments of a split Petri dish (100 × 15 mm). The agar plate was sealed with “Parafilm M” post-inoculation. With a total height of the Petri dish of 15 mm and a 75 cm^2^ surface area, the thickness of the PDA was ~13.33 mm, leaving an ~1.67 mm gap for the fungal growth layer and/or gaseous headspace. To minimize latent growth while in transit, the setup was fully assembled before inoculation of the medium according to manufacturer directions for the KWIK-STIK™ product. A block chart of the experimental flight-hardware setup is given in Figure 1; further details are provided in Supplementary Information 1, Section B.

### Vetting for Cold-Stow

The response of *C. sphaerospermum* CBS 2 to cold storage was determined in a preliminary experiment. A total of six Petri dishes containing PDA were inoculated with the fungus; five were stored at 4°C, with one kept at room temperature (RT) as a control. Plates were removed sequentially after 1, 5, 10, 15, and 20 days. Fungal growth on each plate was monitored at RT and compared to the control.

### On-Orbit Implementation

The equipment was packaged with the inoculated Petri dish before the flight and accommodated in a sealed 2U CubeLab™ (SpaceTango, 2021) between December 2018 and January 2019. Lead-time before the launch was 2 days in cold-stow. Transition time to LEO was 3 days (~1 week to “power-on” after inoculation)^1^, transported to the ISS (U.S. Destiny Laboratory) in cold storage (4°C) on SpaceX mission CRS-16.

In orbit, the experiment was oriented such that the Petri dish (and radiation sensors) faced away from Earth. Pictures of the Petri dish were taken in 30-m intervals for 576 h, resulting in 1,139 images (13 gaps scattered throughout the runtime). Temperature and humidity were measured on average every ~81 s throughout the 622.5-h run-time of the experiment (the three temperature sensors recorded 27,764 readings each). Radiation was measured incrementally, i.e., counts of ionizing events per recording interval, on average every ~95 s, as well as cumulatively (total counts); 23,607 radiation and noise counts were recorded by each radiation sensor. The stand-alone lab was active aboard the ISS for ~26 days, with data downlinked on average every 2–3 days (10 data packages in total) before data collection stopped. The experiment awaited its return to Earth in June 2019. All the collected data can be found in Supplementary Information 2, where the temperature profiles are rendered under “temp” and the recorded radiation data are compiled under “rad” (“g_cont” and “g_exp” are the incremental data, whereas “g_cont_t” and “g_exp_t” are the cumulative data; columns with the suffix “n” in the nametag are noise-counts); growth-data from the on-orbit experiment was merged with the ground control growth-data and can be found under “growth.”

### Ground Controls

In addition to the preflight growth test and integrated on-orbit negative control, Earth-based growth experiments were performed post-flight, replicating the conditions of the flight experiment without radiation. The same methods and techniques were applied when preparing the cultures on a solid medium. As a means of ground control, a time-dependent temperature profile analogous to the on-orbit experiment was replicated, and graphical data were collected at the same intervals to record the growth behavior.

### Evaluation of Growth

Photographs of the cultures were processed with MATLAB (The MathWorks Inc., Natick, MA, US) (MATLAB, 2020) to derive the average brightness values for congruent subsections of each image (cf. Supplementary Information 1, Section C) as a proxy for biomass formation. The average brightness values were normalized to render the relative optical density (OD) ranging from zero to one (cf. Supplementary Information 2, “growth” for raw and processed data) over time. Exponential regression models (implemented in R, cf. Supplementary Information 1, Section D) allowed specific growth rates “*k*” to be determined. Based on these, a relative difference between *k*_*exp*_ for the on-orbit experiment and the ground control, *k*_*ctrl*_, was estimated.

### Evaluation of On-Orbit Radiation

The average difference of radiation counts from the incremental data recorded by the radiation sensors of the control- and fungus-side were plotted over the runtime of the on-orbit experiment by means of locally estimated scatterplot smoothing (Cleveland et al., 1992). Based on the growth data (relative OD), different phases were defined. Phase 1, the initial phase, was defined as relative OD measures below 50% of the maximum, corresponding to the first 19 h. Phase 2, the growth phase, was defined for relative OD measures between 5 and 95% of the maximum, correspondingly starting 5 h after *t*_0_ = 0 h and ending 46 h after *i*_0_. Phase 3, the stationary phase, comprised data from 200 to 622 h after *t*_0_, corresponding to relative OD measures >99% of the total maximum.

### Validation of Statistical Robustness

Logistic growth curve models were fitted to the relative OD measures from the ground controls and on-orbit experiments to test for differences in the slopes of the curves and hence growth behavior. Differences in radiation counts over time between the experiment and the negative control of the on-orbit experiment were modeled with a set of robust regressions. Data preparation and model specification are outlined in Supplementary Information 1, Section D.

## Results and Discussion

### Pre-flight—Cold-Stow Growth-Test

The “Cold-Stow” experiment showed that for all refrigerated sample plates of *C. sphaerospermum* inoculated on PDA, there was nominal fungal growth immediately upon removal from incubation at 4°C (i.e., no fungal growth prior to *t*_0_) for all trialed timeframes. Furthermore, all samples exhibited similar growth once at ambient temperature, regardless of the time spent in cold storage (data not shown).

### Microbial Growth Advantage On-Orbit

From the point the hardware was powered on aboard the ISS, the temperature rose sharply, starting at 22°C and reaching 30°C within the first day, stabilizing after ~1 week around 31.5 ± 2.4°C for the remaining time of the experiment (cf. Supplementary Information 2, “temperature” for temperature profiles as well as humidity data).

**Figure 2:**
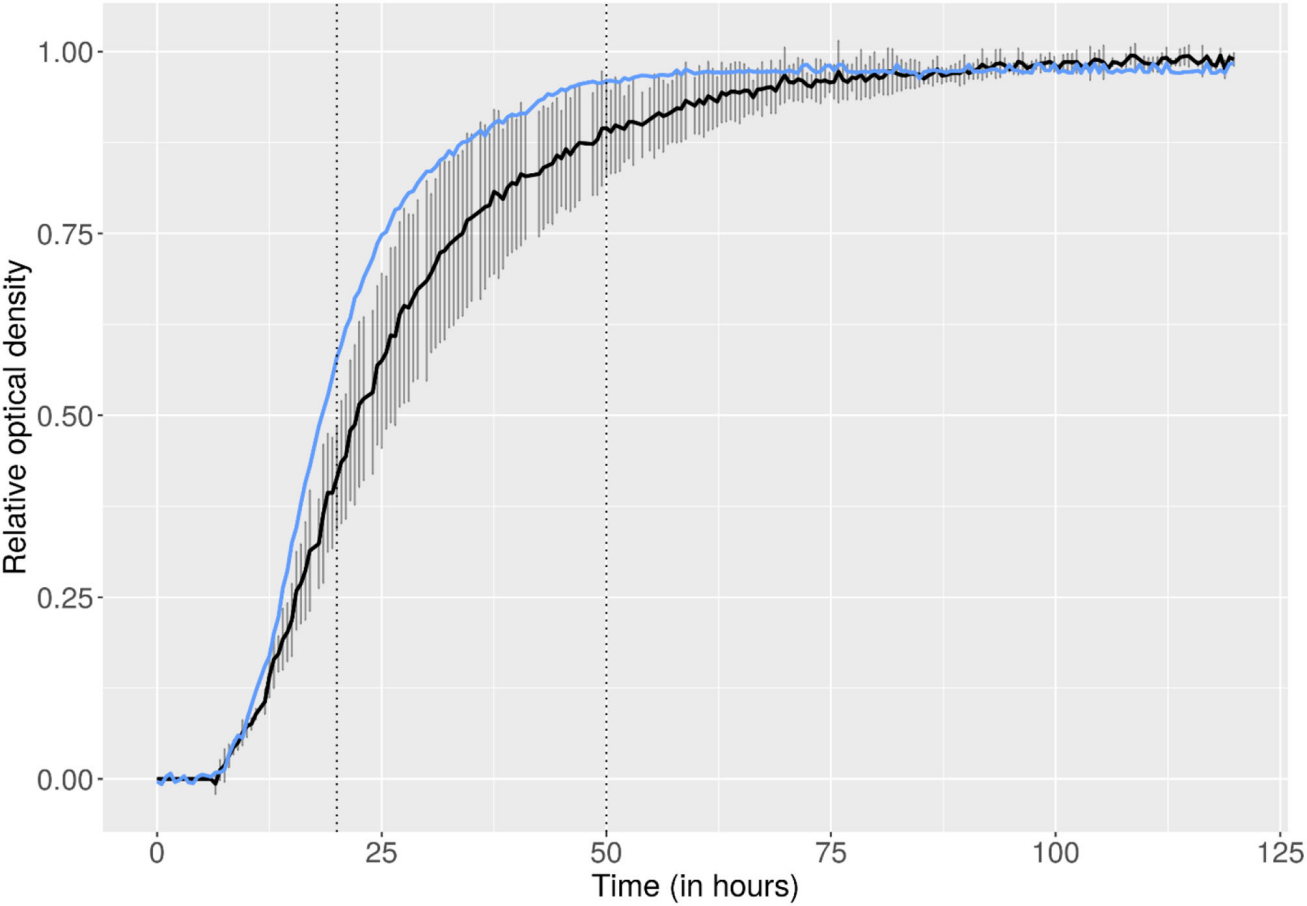
Growth curves (initial 120 h shown) for *C. sphaerospermum* cultivated on solid medium (PDA), depicted by means of connected data points of relative optical densities. While the on-orbit experiment (blue) was a single run, the ground control growth experiment (black) was conducted three times (two replicates in each of the three runs; *t*_0_ was normalized based on the onset of exponential growth of the flight experiment). Error bars show the standard deviation between the three separate runs of the ground control.

Many molds are characterized by slow growth and require up to 2 weeks for significant biomass formation to occur at an optimum temperature of around 25°C (Bosshard, 2011) (only 27 of 50 *Cladosporium* spp. reached growth after 1 week). In the on-orbit lab, *C. sphaerospermum* reached maximum growth and full coverage of the PDA already after 2 days, as discernible from the photographic data (cf. Supplementary Information 1, Section B), as well as the derived growth curve, shown in Figure 2. Comparison with the ground controls indicates that the fungus may have experienced faster-than-normal growth aboard the ISS, as modeled in Supplementary Information 1, Section D. Specifically, the growth rate in the on-orbit experiment was on average 1.21 ± 0.37-times higher (based on *k*_*ground*_1_ = 0.241 h^−1^, *k*_*ground*_2_ = 0.231 h^−1^, *k*_*ground*_3_ = 0.196 h^−1^ and *k*_*flight*_ = 0.299 h^−1^, for the ground controls and on-orbit experiment, respectively, cf. Supplementary Information 1, Section D).

While not immediately numerically tangible, it is worth mentioning that in preliminary ground control experiments, often poor growth was observed at 30°C, as compared to RT (data not shown, not used to establish the growth curves), with only sporadic coverage of the PDA with fungal colonies (cf. Supplementary Information 1, Section B). Only experiments where full coverage of the agar with *C. sphaerospermum* was achieved were selected as representative ground controls; even then a large deviation existed between the three replicates (error bars depicted in Figure 2, cf. also Supplementary Information 2, “growth” for raw-data and additional plots). The observation that higher-than-optimum incubation temperatures on Earth rapidly hindered growth is in accordance with the literature (Zalar et al., 2007). It strengthens the conclusion that space benefited the fungi's overall physiology, where observed growth was strong and homogenous throughout the agar.

The growth advantage in space may be attributable to the stimulating effect of ionizing radiation of the space environment on the fungus, analogous to Earth-based scenarios: when subjected to high levels of γ-radiation (500-times stronger than usual), the metabolism of melanized fungi appears to be significantly increased, with an indication of enhanced growth (Dadachova et al., 2007). Measured by the dose equivalent [144 mSv/a for the ISS and 2.4–6.2 mSv/a for Earth (Cucinotta et al., 2008; ANS, 2020; NRC, 2021)], the radiation on the ISS is about 20- to 60-times stronger than the average background on Earth, however, about 80% of this is attributable to energized particles derived from GCRs and SEPs. Therefore, if radiotrophy was present, the fraction of electromagnetic (γ-) rays utilizable by the fungus may not have been equivalently significant. In addition, particle radiation is vastly more damaging (Cucinotta et al., 2017), which would at least partially negate the beneficial effect. It is plausible that the free-radical scavenging properties of the antioxidant melanin protected the cells against radiation damage, minimizing the detrimental effects inherent to the radiation of the space environment (Cordero et al., 2017; Sharma et al., 2021). The occurrence of radiotrophy, radiotropism, or a radioadaptive response can, however, not be unambiguously proven, as the potential role of microgravity and its impact on fungal growth, whether beneficial or detrimental, cannot be assessed and/or gauged with the employed experimental setup.

While melanization likely confers some degree of radioresistance to many strains of *C. sphaerospermum*, radiotropism or even radiotrophy of all the subspecies of this cosmopolitan fungus is not given, as it may only have evolved in a local subset during radiation exposure in the Chernobyl Exclusion Zone. Nevertheless, different isolates of the mold from the Chernobyl Nuclear Power Plant showed a positive response to irradiation, including a control sample obtained from uncontaminated soil (Dadachova and Casadevall, 2008). This is particularly striking since *C. sphaerospermum* was the only species where this was the case (Zhdanova et al., 2004). Further, enhanced metabolism of *C. sphaerospermum* subjected to ionizing radiation was reported for an unspecified ATCC®-strain^2^ of this species (Dadachova et al., 2007). Therefore, it seems plausible that many *C. sphaerospermum* strains could present radioadaptive and radiostimulative characteristics. It is, however, not a given that this is also valid for the high-energy particle radiation in space, and further in-depth studies are needed to substantiate or refute this. It could also prove worthwhile to study other melanotic micro-fungi in a space environment for indications of radiotropism or radiotrophy, e.g., isolates of *Cladosporium cladosporioides, Aspergillus versicolor, Hormoconis resinae*, or certain *Penicillium* species that have been found to thrive in and around the Chernobyl Nuclear Power Plant (Zhdanova et al., 2004; Dighton et al., 2008), as well as *Wangiella dermatitidis* and *Cryptococcus neoformans*, which have also been investigated for their radioresistance as well as radioadaptation and radiotrophy in related Earth-based studies (Dadachova and Casadevall, 2008; Malo et al., 2019). Subsequent studies could and should also aim to differentiate the impact of microgravity on the (growth) phenotype of these micro-fungi in space. First approaches to studying these molds' responses on a systems-level in the space context have already been made (Singh et al., 2017; Blachowicz et al., 2019).

If radiotrophy continues to prove plausible and appears to be extendable to the radiation of space, it would be tantalizing to aim for the storage of this energy in chemical form as a biotechnological method for ISRU. Actual radiosynthesis, however, remains to be shown, let alone the reduction of carbon compounds into forms with higher energy content or fixation of inorganic carbon driven by ionizing radiation. If, however, radiotrophy is extendable to off-Earth scenarios and the plausibility of radiosynthesis is substantiated, the hypothesis may be established that extraterrestrial life could rely upon analogous mechanisms to harness energy where other forms are unavailable. Black fungi have already been suggested as models for exobiology due to their ability to tolerate extreme physicochemical parameters (Tesei, 2022). With discoveries suggesting the presence of subterranean waterbodies on Mars and icy moons like Enceladus (Witze, 2014; Diez, 2018; Piqueux et al., 2019), this raises the question of whether life may exist within.

### Radiation Measurements in Space

Due to the nature of the employed radiation sensors (PIN photodiode), dosimetric data was not obtained. Nevertheless, approximations can be made regarding the radiation levels (e.g., absorbed dose) to which the experiment was exposed (cf. Supplementary Information 1, Section B). As per the trends of the daily cumulative counts (cf. Supplementary Information 2, “dose” for plots and charts), the periodic fluctuations of the recorded radiation events are correlated with dosimetric data from the U.S. Destiny Laboratory, in particular, those attributed to the transition of the spacecraft through the South Atlantic Anomaly [cf. flight path of the ISS (Lodge-Paolini, 2021)]. Natural phenomena, rather than false measurements, being the explanation for spikes in radiation counts is supported by the observation that these coincide for both sensors throughout the entire experiment, i.e., high radiation events were picked up by both sensors alike. Consequently, this underscores the consistency of the measurements.

### Attenuation of Radiation in the Orbit

**Figure 3:**
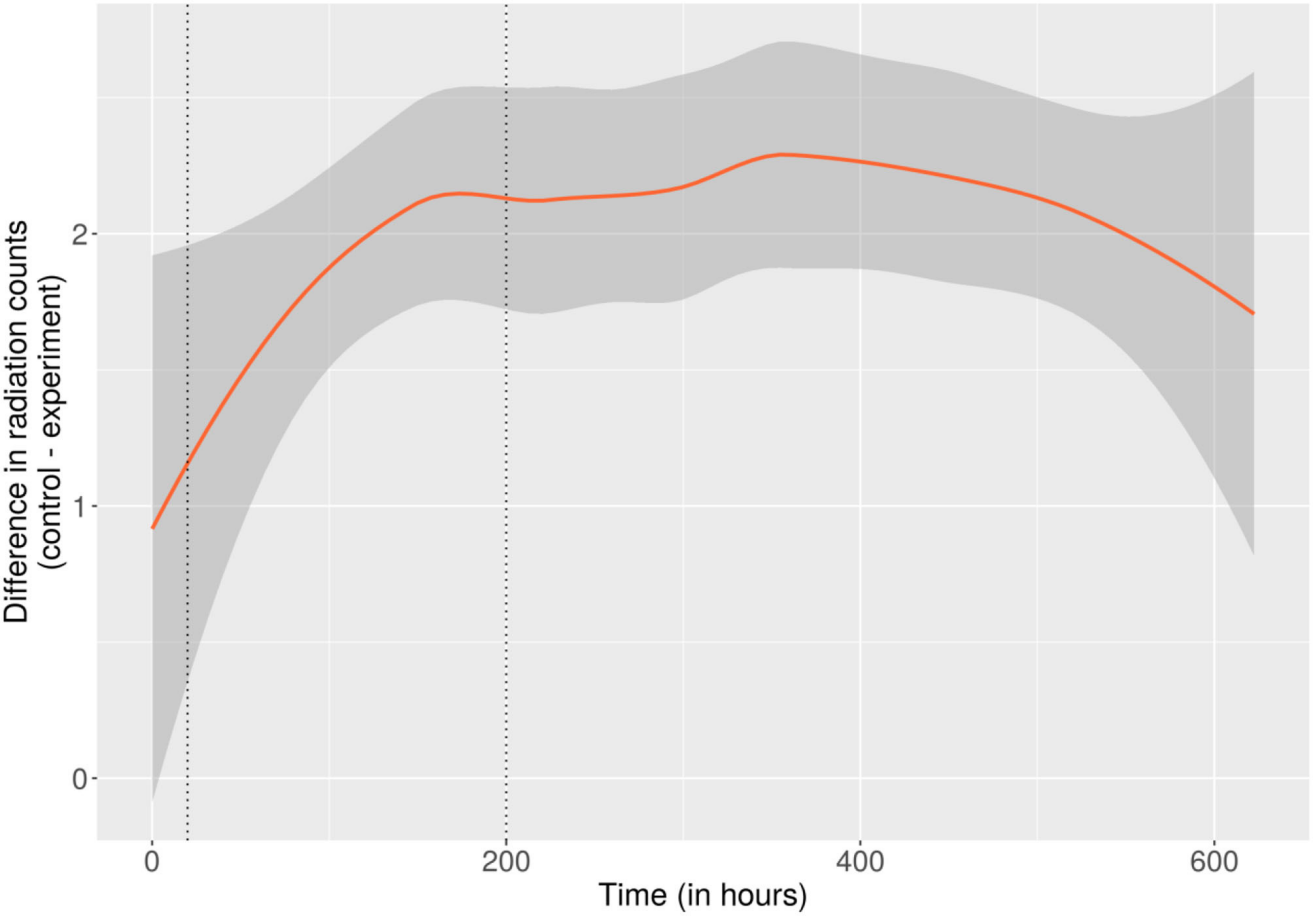
Difference between the incremental radiation counts of the control- and the fungus-side of the on-orbit experiment over the runtime of the experiment. As such, the trendline displays the average difference between the radiation counts measured at intervals of ~1.5 m of locally estimated scatterplot smoothing. The area shaded gray around the trendline corresponds to a 95% confidence band around the smoothed values. Dotted vertical lines were placed for orientation at the time points where growth reached 50% (of the maximum relative OD) and where the formation of biomass and melanin was considered complete (200 h). A more significant difference is apparent toward the later 2/3 of the experiment (200–622 h), where biomass and melanin content were presumably constant, potentially caused by attenuation of the transmitted radiation. The full dataset and additional plots (incremental and cumulative) of the complete radiation data over the whole course of the experiment can be found in Supplementary Information 2, under “rad”.

Independent of the absolute radiation levels, only the relative difference in ionizing events recorded beneath each side of the Petri dish was significant for the experiment. Compared to the negative control, fewer radiation counts were recorded directly beneath the side of the PDA populated with *C. sphaerospermum*, total as well as per minute (147 vs. 151 CPM, cf. Supplementary Information 2, “rad,” columns “g_cont_t” and “g_exp_t”), over the runtime of the experiment. To rule out that this apparent difference was only an effect of, e.g., the radiation sensors' base-deviation, a change in the relative difference between the CPM of the experiment and negative control throughout the experiment that correlates with biomass formation is necessary. As per Figure 3, which displays the average difference of incremental radiation counts using locally estimated scatterplot smoothing, a more significant difference in radiation counts was observable in the late stage of the experiment with full fungal growth compared to the initial hours when minimal biomass existed.^3^ This apparent relationship between the amount of fungal biomass (and putatively the melanin content) and the change in recorded radiation events could indicate attenuation of ionizing radiation. Since only one side of the radiation sensors was protected by the fungus, it is postulated that also only half of the radiation was blocked. Considering the comparatively thin layer of biomass, this may indicate a profound ability of *C. sphaerospermum* to absorb space radiation in the measured spectrum. Another plausible explanation is that conversion of the nutrients contained in the medium into biomass and metabolic end-products and exchange of those with the environment (e.g., consumption of oxygen and production of carbon dioxide) altered the elemental composition of the combined substances in the Petri dish, which resulted in a change in the radiation-blocking properties. However, this theory bears limited potential since the Petri dish and the hardware assembly were sealed.

### Statistical Significance and Robustness

Statistical robustness tests were performed with (A) fungal growth data and (B) radiation attenuation data (as provided in Supplementary Information 2, “growth” and “rad”) with the goal of validating the significance of the drawn conclusions. Specifically, for (A), it was validated that the slope of the growth curve based on the flight-experiment data is steeper than the slope of the growth curves based on the ground control data and that this difference is statistically significant. For (B), it was validated that there is an insignificant difference in the average level of radiation measured beneath the negative control and the fungus/experimental condition in the initial stage of the on-orbit experiment (minimal growth), and the average level of radiation measured beneath the negative control is higher than beneath the fungus/experimental condition in the late stage of the on-orbit experiment (full growth). This supports the hypothesis that the difference in radiation levels beneath the negative control and the fungus/experimental could be attributable to the presence of fungal biomass. A detailed description of the methods, as well as specific results and interpretation of these, can be found in Supplementary Information 1, Section D.

### The Potential of Melanin as a Radioprotector in Space

In radiotrophic fungi, melanin has been proposed as the primary compound that provides radioprotection through dissipation of radiation energy and its absorption through antioxidant activity, as well as direct interaction with the radiation, limiting the generation of free radicals and/or trapping and neutralizing ionized molecules (Cordero, 2017; Pacelli et al., 2017; Malo et al., 2018, 2019). Various natural and (semi-) synthetic melanin-based compounds and composites have been investigated for their ability to block ionizing and non-ionizing radiation and were found to possess significant attenuation capacity (Schweitzer et al., 2009; Cordero et al., 2017; Pombeiro-Sponchiado et al., 2017; El-Bialy et al., 2019; Cao et al., 2020, 2021). For radiation mostly composed of energetic protons or neutrons, substances that are high in hydrogen have the greatest stopping power (Lloyd and Townsend, 2018). As live biomass commonly has a high water content [~60%, (BioNumbers, 2010)], melanized microbes may present an excellent passive shield, particularly for GCR. However, this will require extensive further theoretical and experimental studies (cf. Supplementary Information 1, Section B).

Nevertheless, regardless of how effective a radiation attenuator may be, passive shielding against GCR is ultimately limited by mass density (Dartnell et al., 2007; Chancellor et al., 2018). As a biological compound, natural melanin may be readily available through ISRU and producible by means of biotechnology (Martínez et al., 2019). To increase density, as well as for structural purposes, fungal biomass or melanin itself could be integrated with *in situ* resources that are abundant at the destination, such as regolith (Simonsen et al., 1991), analogous to the concept of Martian “biolith” (Shiwei et al., 2020). In a different application, layers of melanin could be applied to components, such as EVA-suites or inflatable spacecraft and infrastructure, as a constituent of fibrous composites (Blachowicz and Ehrmann, 2021), e.g., in textiles or as resin, to protect them from the surface damage caused by ultraviolet light.

### The Potential of Fungi for ISRU in Space

Leveraging existing resources at outposts on moons and other planets to increase the availability and durability of structural components reduces the required haul of construction material and/or prefabricated elements from Earth. Besides cost savings by reducing up-mass, independence from a supply chain also improves redundancy and robustness of mission-design scenarios. To that end, a multitude of different approaches exists, many of which are still inhibited by the extent of their initially required critical hardware (Gruenwald, 2014; Cichan et al., 2017). Autonomous 3D printing of infrastructure from composites of regolith, for example, has been proposed and demonstrated (MIS, 2020). However, this approach still requires major auxiliary equipment and raw materials for the binding resin. If all materials could be derived or produced on-site, additive manufacturing may become immediately more applicable.

Due to the saprotrophic nature of many fungi, these microorganisms are able to utilize a breadth of carbon compounds and biomass for growth; on Mars, this could be cyanobacterial lysate and/or organic waste, both of which have previously been proposed as substrates for biotechnology (Verseux et al., 2016; Billi et al., 2021). While proven to help produce raw materials (Cockell et al., 2020; Santomartino et al., 2022) and feedstocks for consumables and durable goods (Averesch and Rothschild, 2019), biomanufacturing and bioprocess engineering for space applications are still in the early stages of development (Berliner et al., 2021a,b). Nevertheless, fungi have already been investigated by NASA as a potentially enabling technology to obtain structural components through the application of mycotecture (Rothschild et al., 2019). Expanding the range of organisms being tested for growth on regolith would validate the feasibility of employing melanotic fungi to form composites with *in situ* resources. 3D-bioprinting has already been shown to be feasible with fungal mycelium (Krassenstein, 2014) – advanced additive manufacturing technologies may ultimately allow the creation of smart “living composite” materials that are adaptive, self-healing, and largely autonomous (Nguyen et al., 2018). These “Engineered Living Materials” may provide a game-changing solution to obtain and recover resources and energy and allow the tailored autonomous construction of structural and supporting components in remote locations (Rothschild, 2016).

## Conclusion

With a basic experimental setup implemented as a single small payload on the ISS, it could be shown that the dematiaceous fungus *C. sphaerospermum* can be cultivated in space while being subjected to the unique microgravity and radiation environment of LEO. Growth characteristics indicated an advantage of cultivation on-orbit compared to the ground control. This could be associated with increased radiation in space, potentially causing a radioadaptive response of the microbe, as has been suggested in analogous Earth-based studies. Further, monitoring radiation throughout the experiment indicated that the melanized fungal biomass may have radioprotective properties in space.

Harnessing cosmic radiation and storage of the energy in chemical bound form (biomass) offers the unique opportunity to supplement (biobased) ISRU, reducing the risk of intermittency. Being living organisms, micro-fungi self-replicate from microscopic amounts, which could allow significant savings in up-mass. Biotechnology would thus prove to be an invaluable asset to life support and resource management for explorers on future missions to the Moon, Mars, and beyond.

## Data Availability Statement

The original contributions presented in the study are included in the article/Supplementary Material, and further inquiries can be directed to the corresponding author/s.

## Author Contributions

GS and colleagues of Team Orion conceived of the idea for the study in 2018 and composed the funding proposal. Based on the initial results, GS and colleagues compiled a draft report that served as the basis for the manuscript. NA joined the team in early 2020, comprehensively analyzed the existing data, conducted supporting experiments, researched additional data, and composed the manuscript with support from GS, which was published as a preprint (Shunk et al., 2020). CK subsequently amended the study with regression models and statistical robustness analyses for Journal submission. All authors have read and approved the final version of the manuscript. Correspondence and requests for materials should be addressed to NA.

## Funding

The Higher Orbits Foundation provided funding for the implementation of this project through the Go For Launch! program. NA was partially supported under NASA grant or cooperative agreement award number NNX17AJ31G.

## Conflict of Interest

The authors declare that the research was conducted in the absence of any commercial or financial relationships that could be construed as a potential conflict of interest.

## Publisher's Note

All claims expressed in this article are solely those of the authors and do not necessarily represent those of their affiliated organizations, or those of the publisher, the editors and the reviewers. Any product that may be evaluated in this article, or claim that may be made by its manufacturer, is not guaranteed or endorsed by the publisher.

## Footnotes

^1^launch: 5/12/2018, 18:16 UTC; berthing: 8/12/2018, 15:36 UTC; power on: 9/12/2018 12:54 UTC.

^2^ATCC® currently lists 16 strains of the species *Cladosporium sphaerospermum* in their collection, none of which appear to have been obtained from Ukraine or derived from one of the original Chernobyl isolates.

^3^Although no further gain in optical density was observable later than two days into the experiment, an additional week of maturation was allowed before fungal growth was considered complete and metabolism stationary.

## Abbreviations

CPM: counts per minute
GCR: Galactic Cosmic Radiation
GCRs: Galactic Cosmic Rays
HZE: high atomic number and energy
ISRU: *in situ* resource utilization
ISS: International Space Station
LEO: low Earth orbit
PDA: potato dextrose agar
RT: room temperature.
