# Supplementary material for: Cultivation of the Dematiaceous Fungus Cladosporium sphaerospermum Aboard the International Space Station and Effects of Ionizing Radiation
Source: Front Microbiol. 2022 Jul 5;13:877625. doi: 10.3389/fmicb.2022.877625 (PMC9294542; doi:10.3389/fmicb.2022.877625)
Supplement: Supplementary file 2 [file Data_Sheet_1.PDF]

Supplementary to: "A Self-Replicating Radiation-Shield for Human Deep-Space Exploration: Radiotrophic Fungi can Attenuate Ionizing Radiation aboard the International Space Station"

A. Details on Employed Microorganism

The dematiaceous microscopic fungus *Cladosporium sphaerospermum* is a saprotrophic mold, closely related to 'black yeast'. Many of its subspecies are xerotolerant and halotolerant; in radioactive environments some strains exhibit a radioadaptive response and/or positive radiotropism/radiotrophy. This phenomenon was first noticed when the mold was found to thrive in abundance at the damaged fourth block of the Chernobyl Nuclear Power Plant (Zhdanova et al., 2000). Investigating further, isolates of the microbe from a highly radioactive area were observed to grow towards a source of radiation, another indication for the affinity of the organism for ionizing radiation (Zhdanova et al., 2004). Unexpectedly, also a control-isolate from non-radioactive soil showed the same behavior, which may indicate that radiotrophy is a general feature of this cosmopolitan species. Further, exposure of a *C. sphaerospermum* to radiation levels approximately 500-times higher than background level appeared to stimulate the fungus' growth (Dadachova et al., 2007). In the respective study, a strain obtained from the repository of ATCC® was used (which appears to be unrelated to the isolates from Chernobyl as these are not available through ATCC®). This may further support the theory that radiotrophy is a general feature of this species. Therefore, and because none of the isolates from Chernobyl were easily accessible, *Cladosporium sphaerospermum* Penzig 11289™ (CBS 2), was used in the present study, as it was conveniently available in form of a standardized "KWIK-STIK™" through Microbiologics®.

C. sphaerospermum is viewed as a candidate to potentially perform radiosynthesis, a process which is conceived to be potentially analogous to photosynthesis. However, while the intricacies of photosynthesis have been elucidated and are generally accepted, the feasibility of radiosynthesis remains debated and the potential mechanism obscure. Nevertheless, the ability of certain microscopic fungi to perform melanogenesis is most frequently seen as a prerequisite for radioadaptation and it has been suggested that ionizing radiation alters the chemical properties of the cellular pigment melanin (Dadachova and Casadevall, 2008). Under high levels of ionizing electromagnetic radiation this putatively leads to increased rates of electron-transfer to metabolism, theoretically allowing for a net energy-gain, to be used for physiological functions and/or to reduce carbon (Turick et al., 2011) (analogous to photosynthesis, where chlorophyll captures the energy of non-ionizing electromagnetic radiation, i.e. light). Unlike melanocytes in humans, in dematiaceous fungi the melanin is crystallized on the cell-walls (Eisenman and Casadevall, 2012). The radioresistance this compound confers is better known and understood than its implications relating to radiotrophy: melanin provides protection of the cells from oxidizing agents as scavenger of free radicals, which are generated by different forms of ionizing (e.g., γ- and X-rays) as well as non-ionizing radiation (e.g., UV-light), giving the mold an ecological advantage in extreme environments on Earth, natural as well as non-natural. The expectation is that the whole of these capabilities may also prove advantageous in the high-radiation environment outside of Earth's protective magnetosphere (Cordero, 2017;Pacelli et al., 2017).

B. Specifics of Experimental Hardware

Absorption Spectrum of Radiation Sensors

Ionizing cosmic radiation, or in other words radiation with enough kinetic or electromagnetic energy to strip electrons from an atom, comes in two forms (excluding photons from the classification): wave radiation (electromagnetic waves) and particle radiation, like e.g. α- and β-particles, energetic protons or HZE ions (Simpson, 1983). In the electromagnetic spectrum, ionizing (wave-) radiation is characterized by energies ranging from a few hundred eV to about 1 MeV but can also be much higher for cosmic radiation. The PocketGeiger Type5 is designed to measure wave radiation, specifically of X- and γ-type (Radiation-Watch, 2011): at 23°C the PIN photodiode of the X100-7 SMD (First Sensor AG, Berlin, Germany) is particularly sensitive for γ-radiation with energies of 3-20 keV, with highest absorbance between 5.5 keV and 10 keV (FirstSensor, 2013).

Levels and Energy of Measured Radiation

Based on the total cumulative recorded radiation counts over the 622.5 hours of the experiment, an average of 149 CPM (counts-per-minute) was determined. With reported radiation levels for the ISS of ≈ 144 mSv/a (≙ 0.274 µSv/min) or 58 mGy/a (≙ 0.11 µGy/min) (Cucinotta et al., 2008), respectively, a single count thus corresponds to an average of approx. 5.6 nSv or 2.2 nGy, respectively.

To more specifically inform the type (and energy) of radiation measured by the PocketGeiger Type5, daily dosage data obtained from the ISS (U.S. Destiny Laboratory) for the time of the experiment was correlated with the radiation counts from the experiment (cf. supplementary information 2, 'dosage'). The trend followed most closely the fraction of the radiation attributed to the South Atlantic Anomaly (SAA), while no direct correlation was observed between the radiation counts and dosage attributed to Galactic Cosmic Radiation (GCR). This indicates that, in addition to secondary radiation in their specific absorbance range, electrons in the range of hundreds of keV and energetic protons with energies exceeding 100 MeV, as are typical for the inner Van Allen belt, where likely the primary forms of energetic particles that caused signals which were picked up by the employed radiation sensors. Based on that conclusion, one CPM would correspond to approx. 0.5 nGy.

Supplementary to: “A Self-Replicating Radiation-Shield for Human Deep-Space Exploration: Radiotrophic Fungi can Attenuate Ionizing Radiation aboard the International Space Station”

<u>Assessment of Radiation Attenuation Capacity</u>

Due to the complex nature of cosmic radiation and vicissitude of Space weather, it is challenging to replicate Space radiation in Earth-based laboratories to study the shielding properties of materials. The same reasons make the accurately theoretical description of the radiation in Space equally difficult. Nevertheless, tools like OLTARIS (Singleterry et al., 2011) and SPENVIS (ESA, 2020) and/or Monte Carlo simulations (Issa et al., 2018) (using platforms like e.g. GEANT4 and CREME96) (Falzetta et al., 2008) are sensible methods to model the interaction of energized particles with matter. Such *in silico* analyses go hand-in-hand with experimental studies, as are for example possible by means of the Galactic Cosmic Ray Simulation (GCRSim) (BNL, 2020), as well as a deeper understanding of the health-risk that the radiation in Space poses for human crews. While such analyses are out of the scope of this study, we feel it is nevertheless important to mention and acknowledge such methods, to identify the challenges that exist for maturation of the here described technology and give a perspective of how to approach these in follow-up studies.

<u>Flight Hardware and Experimental Implementation</u>

The flight-hardware was packaged as a 2U (double standard-size) CubeLab™ (sealed) module (4”×4”×8”), which has a volume of 103.4 in³, is air-tight, and provides up to 20 W of power. The unit was hosted in the U.S. Destiny Laboratory of the ISS. The assembled unit is shown in figure S1.

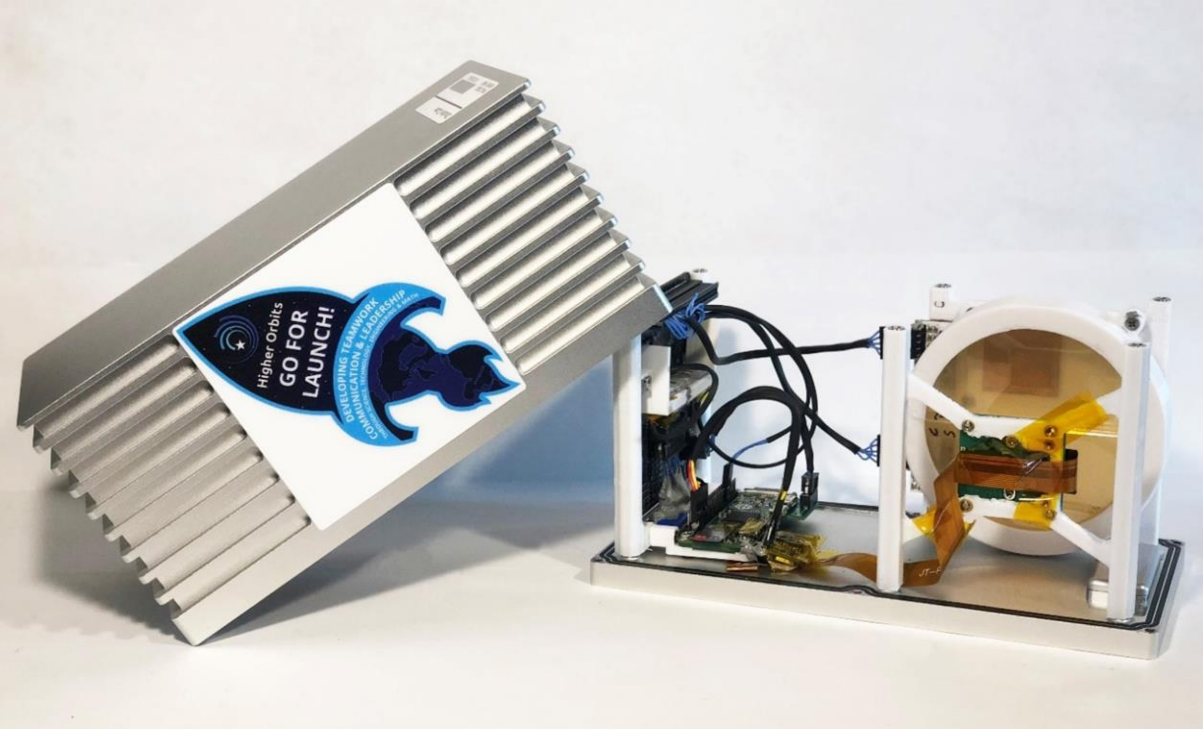

Figure S1*:* Fabricated flight hardware unit, packaged in a 2U Space Tango CubeLab™. Media Courtesy: Space Tango, Inc.

C. Methodology for Evaluation of Microbial Growth

Growth of *C. sphaerospermum* was characterized based on the relative brightness of the time-series photos of the fungus grown on solid medium (potato-dextrose-agar). For this purpose, a representative section of the fungus-covered agar with high contrast was chosen, as indicated in figure S2 I for the on-orbit experiment, and the average brightness of the image-section was determined. MATLAB was utilized to automate evaluation of the images by means of a script based on the function "HSV colormap array". All photos were processed identically using the same picture-coordinates. The obtained values were standardized to an optical density (OD) of 0 at t_0 and normalized to the average brightness value of the last 4-8 hours of the experiment to a maximum OD of 1. Plotted over time, a relative growth-curve was thus obtained (cf. supplementary information 2, 'growth').

The data for the ground-control experiments were processed equivalently, while integrating multiple replicates into one growth-curve. Specifically, three individual ground-control experiments were carried out (separate runs), while each experiment contained three replicates (separate Petri dishes), the OD-data thereof was combined to obtain robust growth-curves with standard deviation for each experiment (cf. supplementary information 2, 'growth').

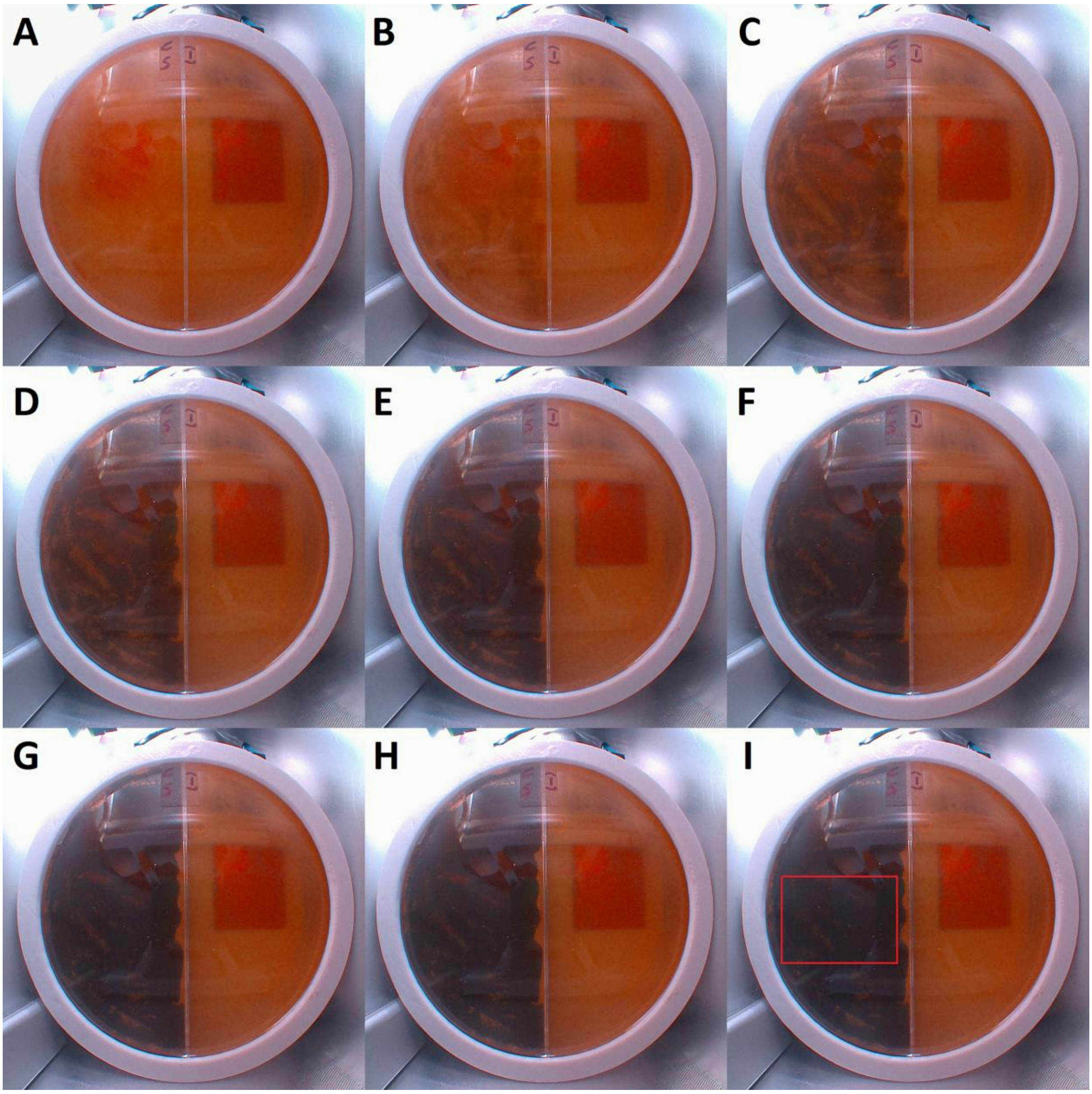

Figure S2: Photographic data of fungal growth. Images A - I of the on-orbit experiment show fungal growth on the agar of one side of the split Petri dish in intervals of 6 hours starting with A: $t_0 = 0$ hours continuing until I: t = 48 hours. The red frame in the last picture (I) indicates the section of the picture used to derive the growth data by means of picture brightness, exemplary for all pictures.

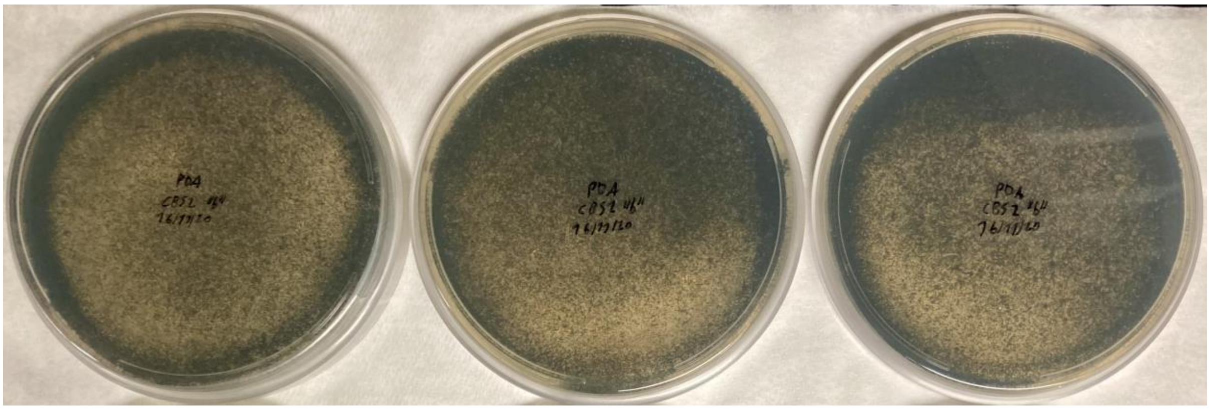

Figure S3: Photo of ground-control experiment, showing growth at 30°C 14 days after inoculation.

D. Statistical Analysis of Acquired Data

Materials and Methods

The statistical analyses were based on two combined datasets, created from the consolidated data in supplementary information 2. Dataset 1 included fungal growth data of the first 45 hours from the respective onsets of fungal growth, as per the relative optical densities (ODs) from the on-orbit experiment and the three ground-controls. The on-orbit experiment and ground-control data, with growth data recorded in intervals of 30 min, were merged by fuzzy-matching the relative ODs to their closest neighbors in time. Dataset 2 comprised radiation measures from the on-orbit experiment for the experimental condition and negative-control covering the full runtime of 622.5 hours.

The relative OD data of the on-orbit experiment were used to define three phases: Phase 1, the initial phase, was defined for relative ODs below 50% of the maximum (i.e. OD < 0.5), corresponding to the first 19 hours. Phase 2, the growth-phase, was defined based on exponential character of increasing OD, which was the case roughly between 5 and 15 hours (cf. figure S5). Phase 3, the stationary phase, comprised data from 200 until 622.5 hours after t_0, corresponding to relative ODs greater than 99% of the total maximum (i.e. 0.99 < OD).

The growth data from the on-orbit experiment and the ground-controls were compared by fitting exponential and logistic curves to the relative OD measures of dataset 1. Specifically, we model: $growth = asym\ /\ (1 + exp((xmid - hour)/scale))$ where the outcome variable fungal *growth* (relative OD) is modeled as a function of time in hours with *asym* being the higher asymptote, *xmid* the time value at the inflection point of the curve and *scale* the inverse of the slope at the inflection point. We estimated joint models with the on-orbit experiment and ground-control data in order to test for differences in the slope of the logistic curves.

In order to account for spikes/outliers in the radiation data, we used robust statistical methods to analyze the relationship between the two conditions (experimental condition vs. negative-control) and radiation attenuation using dataset 2. We utilized robust regression models using the M-estimator where outlier values are penalized (down-weighted) in the estimation process (Huber, 1981). Specifically, in phase 1 and 3 robust regressions were used to estimate: $g_{it^{(p)}} = \alpha_0 + \alpha_1 exp_{it^{(p)}} + \beta' x_{it^{(p)}} + \varepsilon_{it^{(p)}}$ where the outcome variable $g_{it^{(p)}}$ represents radiation counts for condition i (experimental condition, negative-control), reading $t^{(p)}$ and phase p, α_0 is the baseline radiation (constant), α_1 the coefficient for the indicator variable $exp_{it^{(p)}}$ (experimental condition = 1, negative-control = 0), β is a vector of coefficients for auxiliary variables $x_{it^{(p)}}$ (including time in hours and $exp_{it^{(p)}} \times hour_{t^{(p)}}$) and $\varepsilon_{it^{(p)}}$ is an error term. We report heteroskedasticity-robust standard errors for the coefficients of all robust regression models (MacKinnon and White, 1985). Both model sets are accompanied by Wilcoxon rank sum tests for location shifts as an alternative non-parametric approach to test for differences in radiation attenuation between the two conditions (Hollander et al., 2015).

Supplementary to: "A Self-Replicating Radiation-Shield for Human Deep-Space Exploration: Radiotrophic Fungi can Attenuate Ionizing Radiation aboard the International Space Station"

Assumptions and Hypothesis

We tested the following hypotheses:

1) H1: The slope of the growth-curve based on the on-orbit experiment data is steeper compared to the growth based on the ground-control data
2) H2: In the beginning (phase 1) there is insignificant difference in the average level of radiation measured beneath the negative control and the fungus / experimental condition
3) H3: In the end (phase 3) the average level of radiation measured beneath the negative-control is significantly higher than beneath the fungus / experimental condition

Software

R (Version 4.0.2) (Team, 2020) with packages fuzzyjoin (0.1.6) (Robinson, 2020), nlme (3.1-149) (Pinheiro et al., 2020), MASS (7.3-53) (Venables and Ripley, 2002), lmtest (0.9-38) (Zeileis and Hothorn, 2002), sandwich (3.0-0) (Zeileis, 2004).

Code

Scripts utilized to conduct the analyses described herein are made available through GitHub at: https://github.com/chkern/space-radiation

Results and Discussion

We start by presenting growth-curve models that were fitted to the relative ODs (representative of *growth*) of the on-orbit experiment and the ground-controls of dataset 1. In the exponential phases of the ground-controls we observed $growth_{ground} = 0.033 \times exp(0.226 \times hour)$ with the standard error of the growth-rate (0.226 h^{-1}) being ±0.014 h^{-1}, based on a joint model of the average relative ODs of the three ground-control growth-curves. In the exponential growth-phase of the on-orbit experiment (5 to 15 hours after t_0) we observe $growth_{flight} = 0.017 \times exp(0.299 \times hour)$, i.e. a growth-rate of 0.299 h^{-1} with a standard error of ±0.015 h^{-1}. The ODs with the fitted exponential growth-curves are shown in figure S4.

Table S1 shows coefficients (higher asymptote, midpoint, scale) of logistic growth-curves $asym \,/\, (1 + exp(b(xmid - hour)))$ that were fitted to the combined flight and ground-control data for the full timeframe of dataset 1 (45 hours from the onset of fungal growth). Specifically, model 1.1 is based on the on-orbit experiment and the mean of the average relative ODs of the three ground-controls, while models 1.2, 1.3, and 1.4 compare the on-orbit experiment data to the average relative OD of each ground-control experiment (1 to 3), respectively. In order to identify differences in fungal growth, the curve models allow for different slopes (b = 1/*scale*) between on-orbit experiment and ground-control and, in addition, for a shift of the inflection point of the curve (*xmid*). In line with our expectations (H1), we observe a steeper slope of the logistic curves of the flight experiment, as

compared to both, the average of the ground-controls (model 1.1: $p \leq 0.001$) as well as the individual runs (model 1.2: $p = 0.042$, model 1.3: $p \leq 0.001$, model 1.4: $p \leq 0.001$). In model 1.1 the slope b of the logistic curve based on the on-orbit experiment is $1/scale_{flight}$= 0.252 h^{-1}, whereas the slope of the ground-controls' average is $1/(scale_{flight} + scale_{ground-flight})$= 0.16 h^{-1}. Based on models 1.2, 1.3 and 1.4, the individual slopes for ground-control 1, 2 and 3 are 0.215 h^{-1}, 0.156 h^{-1} and 0.118 h^{-1} (corresponding to a 1.17-fold, 1.615-fold or 2.135-fold growth-rate of the on-orbit experiment, as compared to each ground-control, respectively). Hence, the average growth-rate advantage of the on-orbit experiment can be stated as 1.64-fold with a standard error of ± 0.279 and a standard deviation of ± 0.483. The fitted logistic growth-curves are shown in figure S5.

Turning to dataset 2 and H2, table S2 presents robust regressions that model the incremental radiation counts in the initial phase of the flight experiment as a function of the experimental condition indicator (model 2.1), including time (model 2.2) and an interaction term between time and experimental condition (model 2.3) as covariates. Following our expectations (H2), we observe no difference ($p = 0.970$) in the average level of radiation between the two conditions in phase 1, i.e., at the onset of fungal growth. This holds true for all model specifications and is supported by the first Wilcoxon rank sum test as shown in table S3.

Table S4 shows robust regressions that model the incremental radiation counts in the stationary phase, with the experimental condition indicator being the main predictor of interest (model 3.1), and time (model 3.2) and an interaction term between time and experimental condition (model 3.3) as covariates. In line with the expectation (H3), a difference ($p = 0.069$) in radiation levels between both conditions can be observed in model 3.1, with lower radiation beneath the side of the petri dish populated with fungal biomass. This finding is confirmed when controlling for time (model 3.2) and further supported by the second Wilcoxon rank sum test in table S3. Lastly, model 3.3 shows no indication of a time-varying effect of the experimental condition indicator on radiation levels in the stationary phase.

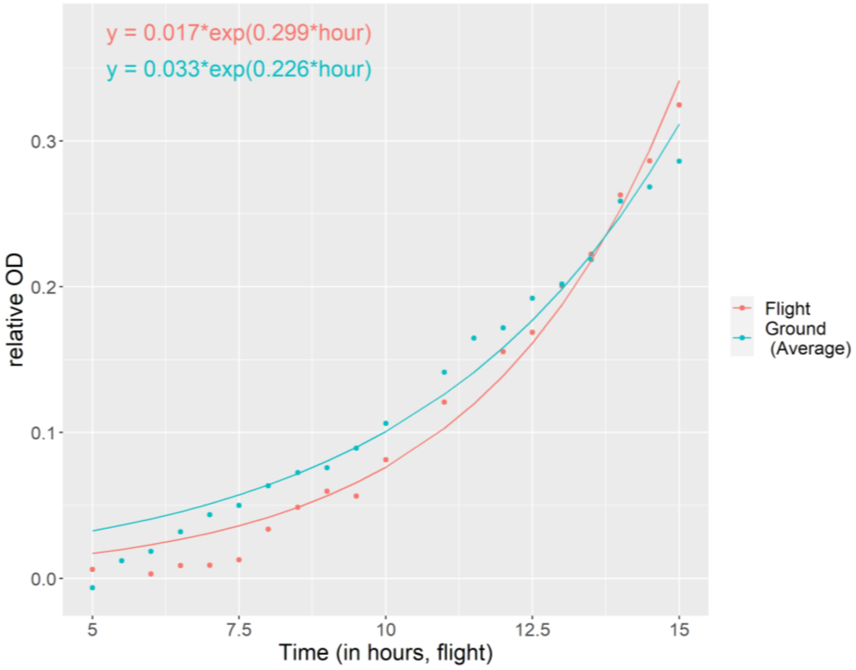

Figure S4: Relative OD readings and growth-curves for the exponential growth-phases of the on-orbit experiment and ground-controls. The equations of the exponential regressions were used to approximate the growth-rates of the fungus (k_{flight} = 0.299 h^{-1} and k_{ground} = 0.226 h^{-1}).

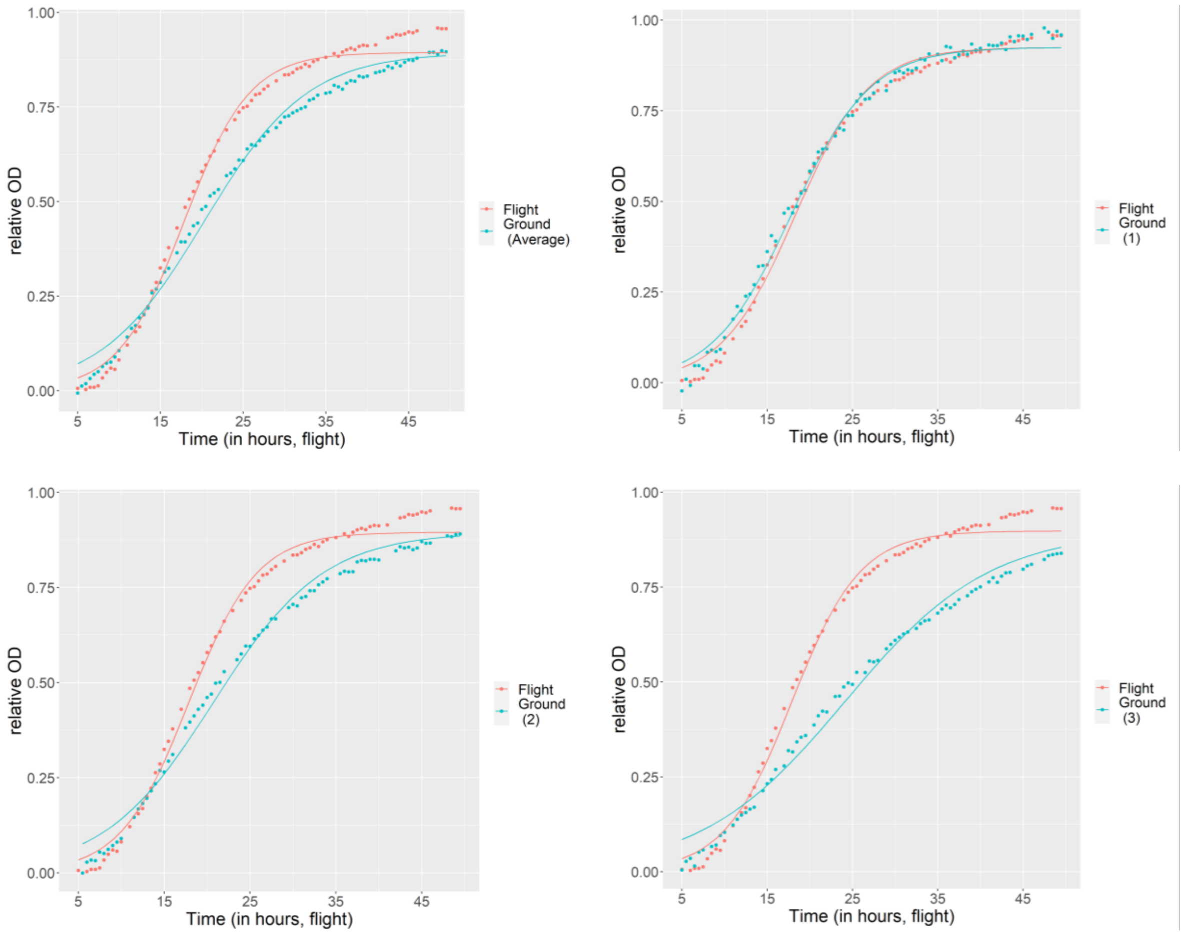

Figure S5: Relative OD readings and logistic growth-curves for the on-orbit experiment and ground-control (average): model 1.1, (1): model 1.2, (2): model 1.3, (3): model 1.4.

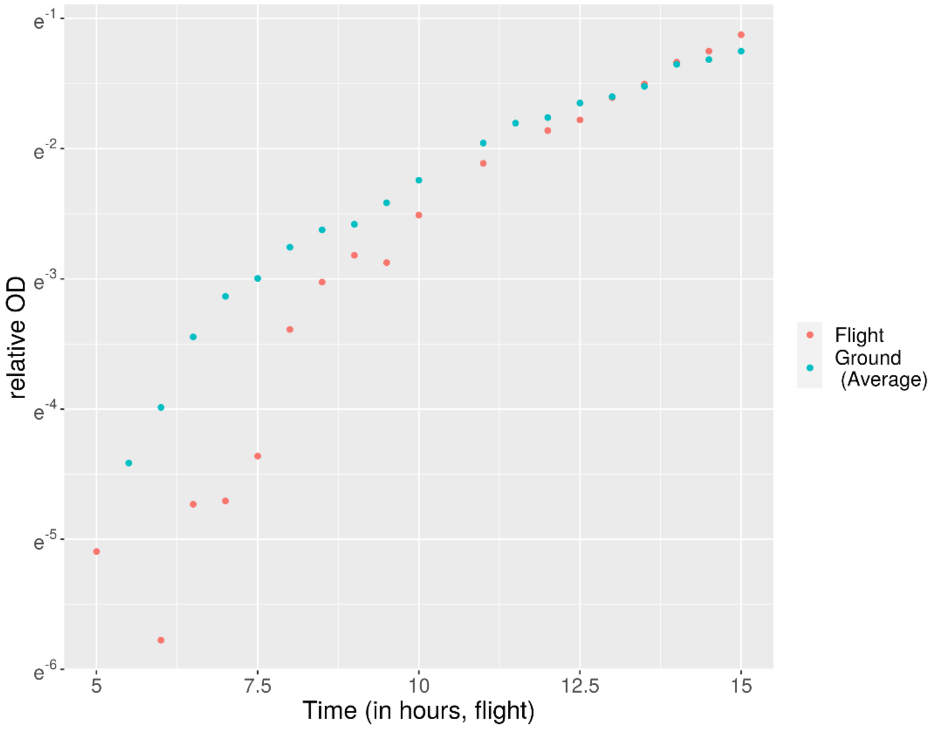

Figure S6: Semi-logarithmic plot of relative OD readings and growth-curves for the exponential growth-phases of the on-orbit experiment and ground-controls (complementing figure S4).

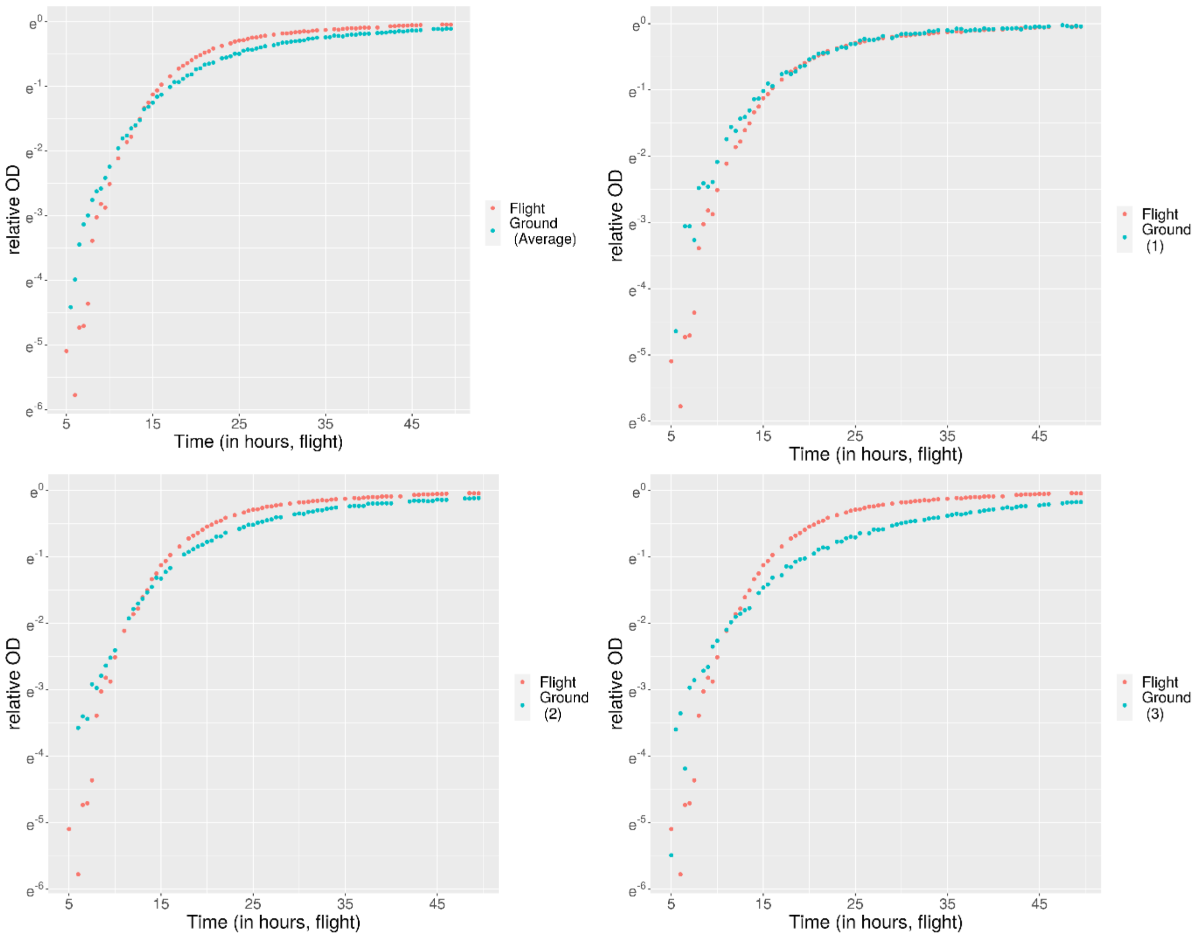

Figure S7: Semi-logarithmic plot of relative OD readings and logistic growth-curves for the on-orbit experiment and ground-control (average): model 1.1, (1): model 1.2, (2): model 1.3, (3): model 1.4.

Table S1: Logistic growth-curves (**phase 1** and **2**, outcome: relative OD)

model	(1.1)	(1.2)	(1.3)	(1.4)
asym	0.894	0.925	0.895	0.898
se	(0.005)	(0.004)	(0.005)	(0.006)
xmid (flight) [hour]	12.847	13.302	12.863	12.902
se	(0.141)	(0.133)	(0.144)	(0.154)
xmid (ground – flight) [hour]	2.417	-0.482	2.859	6.238
se	(0.200)	(0.169)	(0.211)	(0.248)
p	0.000000	0.005	0.000000	0.000000
scale (flight) [hour]	3.965	4.330	3.977	4.008
se	(0.124)	(0.117)	(0.126)	(0.134)
scale (ground – flight) [hour]	2.278	0.315	2.431	4.466
se	(0.183)	(0.154)	(0.194)	(0.230)
p	0.000000	0.042	0.000000	0.000000
n	154	154	145	146
AIC	-637.810	-655.788	-596.116	-589.044
BIC	-619.589	-637.566	-578.256	-571.143

Note: we report heteroskedasticity-robust standard errors.

Table S2: Robust regression models (**phase 1**, outcome: g_{it})

model	(2.1)	(2.2)	(2.3)
time [hours]		0.446	0.376
se		(0.149)	(0.218)
p		0.003	0.085
exp	-0.110	-0.011	-0.899
se	(2.853)	(2.812)	(3.998)
p	0.970	0.997	0.823
time × exp			0.136
se			(0.299)
p			0.649
constant	62.629	59.587	60.062
se	(2.065)	(2.482)	(2.884)
n	428	428	428
AIC	4473.898	4479.005	4480.831
BIC	4486.076	4495.241	4501.127

Note: we report heteroskedasticity-robust standard errors.

Table S3: Wilcoxon rank sum tests for location shift in g_t (**phase 1** and **3**)

phase	mean $(g_{exp=1})_t$	median $(g_{exp=1})_t$	mean $(g_{exp=0})_t$	median $(g_{exp=0})_t$	*n*	*W* (test statistic)	*p*
1	67.173	62.5	67.794	60.5	214	22867.5	0.981
3	90.269	58	92.440	59	15918	128174240	0.070

Table S4: Robust regression models (**phase 3**, outcome: g_{it})

model	(3.1)	(3.2)	(3.3)
time [hours]		-0.012	-0.011
se		(0.002)	(0.003)
p		0.000	0.00004
exp	-0.824	-0.823	-0.526
se	(0.452)	(0.451)	(1.588)
p	0.069	0.068	0.741
time × exp			-0.001
se			(0.004)
p			0.845
constant	67.931	72.428	72.279
se	(0.322)	(0.826)	(1.130)
n	31836	31836	31836
AIC	426044.274	426038.782	426040.793
BIC	426069.379	426072.256	426082.635

Note: we report heteroskedasticity-robust standard errors.

E. References

BNL (2020). *NSRL User Guide*. Brookhaven National Laboratory. Available: https://www.bnl.gov/nsrl/userguide/GCRSim.php [Accessed 2020/11].

Cordero, R.J.B. (2017). Melanin for space travel radioprotection. *Environ. Microbiol.* 19**,** 2529-2532.

Cucinotta, F.A., Kim, M.-H.Y., Willingham, V., and George, K.A. (2008). Physical and biological organ dosimetry analysis for international space station astronauts. *Radiat. Res.* 170**,** 127-138.

Dadachova, E., Bryan, R.A., Huang, X., Moadel, T., Schweitzer, A.D., Aisen, P., Nosanchuk, J.D., and Casadevall, A. (2007). Ionizing Radiation Changes the Electronic Properties of Melanin and Enhances the Growth of Melanized Fungi. *PLoS ONE* 2**,** e457.

Dadachova, E., and Casadevall, A. (2008). Ionizing Radiation: how fungi cope, adapt, and exploit with the help of melanin. *Curr. Opin. Microbiol.* 11**,** 525-531.

Eisenman, H.C., and Casadevall, A. (2012). Synthesis and assembly of fungal melanin. *Appl. Microbiol. Biotechnol.* 93**,** 931-940.

ESA (2020). *Space Environment, Effects, and Education System*. SPENVIS. Available: https://www.spenvis.oma.be/ [Accessed 2020/11].

Falzetta, G., Longo, F., and Zanini, A. (2008). "GEANT4 and CREME96 comparison using only proton fluxes", in: *arXiv*. (online: arXiv.org).

FirstSensor (2013). *Part Description X100-7 SMD*. Berlin, Germany: First Sensor AG. Available: https://cdn.sparkfun.com/assets/learn_tutorials/1/4/3/X100-7_SMD_501401.pdf
https://www.mouser.com/catalog/specsheets/x100-7-smd-501401-prelim%5B1%5D.pdf [Accessed 2020/11].

Hollander, M., Wolfe, D.A., and Chicken, E. (2015). *Nonparametric Statistical Methods* New York: John Wiley and Sons.

Huber, P.J. (1981). *Robust Statistics.* New York: John Wiley and Sons.

Issa, S.a.M., Saddeek, Y.B., Tekin, H.O., Sayyed, M.I., and Shaaban, K.S. (2018). Investigations of radiation shielding using Monte Carlo method and elastic properties of PbO-SiO2-B2O3-Na2O glasses. *Curr. Appl. Phys.* 18**,** 717-727.

Mackinnon, J.G., and White, H. (1985). Some heteroskedasticity-consistent covariance matrix estimators with improved finite sample properties. *J. Econom.* 29**,** 305-325.

Pacelli, C., Bryan, R.A., Onofri, S., Selbmann, L., Shuryak, I., and Dadachova, E. (2017). Melanin is effective in protecting fast and slow growing fungi from various types of ionizing radiation. *Environ. Microbiol.* 19**,** 1612-1624.

Pinheiro, J., Bates, D., Debroy, S., Sarkar, D., and Team, R.C. (2020). *nlme: Linear and Nonlinear Mixed Effects Models*, CRAN. Available: https://CRAN.R-project.org/package=nlme [Accessed 10/2021].

Radiation-Watch (2011). *RADIATION WATCH: 専門家向け資料*. Radiation-Watch.org. Available: http://www.radiation-watch.org/2011/05/professional.html#emb [Accessed 2020/11].

Robinson, D. (2020). *fuzzyjoin: Join Tables Together on Inexact Matching*, CRAN. Available: https://github.com/dgrtwo/fuzzyjoin [Accessed 10/2021].

Simpson, J.A. (1983). Elemental and Isotopic Composition of the Galactic Cosmic Rays. *Annu. Rev. Nucl. Part.* 33**,** 323-382.

Singleterry, R.C., Blattnig, S.R., Clowdsley, M.S., Qualls, G.D., Sandridge, C.A., Simonsen, L.C., Slaba, T.C., Walker, S.A., Badavi, F.F., Spangler, J.L., Aumann, A.R., Neal Zapp, E., Rutledge, R.D., Lee, K.T., Norman, R.B., and Norbury, J.W. (2011). OLTARIS: On-line tool for the assessment of radiation in space. *Acta Astronaut.* 68**,** 1086-1097.

Team, R.C. (2020). "R: A language and environment for statistical computing". (Vienna, Austria: R Foundation for Statistical Computing).

Turick, C.E., Ekechukwu, A.A., Milliken, C.E., Casadevall, A., and Dadachova, E. (2011). Gamma radiation interacts with melanin to alter its oxidation–reduction potential and results in electric current production. *Bioelectrochem.* 82**,** 69-73.

Venables, W.N., and Ripley, B.D. (2002). *Modern Applied Statistics with S-PLUS.* New York: Springer.

Zeileis, A. (2004). Econometric Computing with HC and HAC Covariance Matrix Estimators. *J. Stat. Softw.* 11**,** 1-17.

Zeileis, A., and Hothorn, T. (2002). Diagnostic Checking in Regression Relationships. *R News* 2**,** 7-10.

Zhdanova, N.N., Tugay, T., Dighton, J., Zheltonozhsky, V., and Mcdermott, P. (2004). Ionizing radiation attracts soil fungi. *Mycol. Res.* 108**,** 1089-1096.

Zhdanova, N.N., Zakharchenko, V.A., Vember, V.V., and Nakonechnaya, L.T. (2000). Fungi from Chernobyl: mycobiota of the inner regions of the containment structures of the damaged nuclear reactor. *Mycological Research* 104**,** 1421-1426.
